# Smaller Intervertebral Disc Volume and More Disc Degeneration after Spinal Distraction in Scoliotic Children

**DOI:** 10.3390/jcm10102124

**Published:** 2021-05-14

**Authors:** Sebastian Lippross, Paul Girmond, Katja A. Lüders, Friederike Austein, Lena Braunschweig, Stefan Lüders, Konstantinos Tsaknakis, Heiko M. Lorenz, Anna K. Hell

**Affiliations:** 1 Department of Trauma, Orthopedic and Plastic Surgery, University Medical Center Goettingen, 37075 Goettingen, Germany; sebastian.lippross@uksh.de (S.L.); katja.lueders@med.uni-goettingen.de (K.A.L.); lena.braunschweig@med.uni-goettingen.de (L.B.); konstantinos.tsaknakis@med.uni-goettingen.de (K.T.); heiko.lorenz@med.uni-goettingen.de (H.M.L.); 2 Department of Orthopedic and Trauma Surgery, University Medical Center Schleswig-Holstein, Campus Kiel, 24105 Kiel, Germany; paul.girmond@gmx.de; 3 Department of Diagnostic and Interventional Neuroradiology, University Medical Center Hamburg-Eppendorf, 20251 Hamburg, Germany; f.austein@uke.de; 4 Mahr GmbH, 37073 Goettingen, Germany; stefan.lueders@mahr.de

**Keywords:** intervertebral disc, volume, disc degeneration, spinal muscular atrophy, scoliosis, growth-friendly implants, MRI

## Abstract

In recent decades, magnetically controlled growing rods (MCGR) were established to treat progressive early-onset scoliosis. The aim of this investigation was to assess the effect of long-term MCGR with continuous distraction on intervertebral discs in scoliotic children. Magnetic resonance imaging (MRI) of 33 children with spinal muscular atrophy was analyzed by grading intervertebral disc degeneration (IDD) and measuring intervertebral disc volume. Cohort I (*n* = 17) were children who had continuous spinal distraction with MCGRs for 5.1 years and MRI before (av. age 8.1) and after (av. age 13.4) MCGR treatment. Cohort II (*n* = 16, av. age 13.7) were patients without prior surgical treatment. Lumbar intervertebral disc volume of cohort I did not change during 5.1 years of MCGR treatment, whereas disc volumes were significantly larger in age- and disease-matched children without prior treatment (cohort II). Cohort I showed more IDD after MCGR treatment in comparison to early MRI studies of the same patients and children without surgical treatment. MRI data showed a volume reduction and disc degeneration of lower thoracic and lumbar intervertebral discs in scoliotic children after continuous spinal distraction with MCGRs. These effects were confirmed in the same subjects before and after treatment as well as in surgically untreated controls.

## 1. Introduction

Growth friendly spinal implants (GFSI) have recently become a standard of care for early onset scoliosis (EOS). There are many reports in the literature supporting the idea of early minimally invasive treatment as an effective alternative to casting, bracing or “watch and wait” strategies [1]. It is a well-known problem in pediatric orthopedics and spinal surgery that early deformities tend to progress quickly, which can ultimately result in thoracic insufficiency syndrome, while spinal fusion, which is the most effective method to control scoliotic curve progression, will lead to the same condition if performed too early. GFSI have, therefore, evolved, awakening the hope of providing an effective therapeutic concept. There are a handful of different constructs used, all based on the same principle: hooks or screws may be used to anchor an expandable rod between pedicles, laminae, ribs and/or the pelvic crest. Lengthening rods may either be magnetically controlled or motorized or conventionally expanded by minor repeated surgical procedures. Regardless of the choice of construct, several studies have demonstrated good deformity control, especially in syndromic, neuromuscular and/or congenital disease [2].

**Figure 1:**
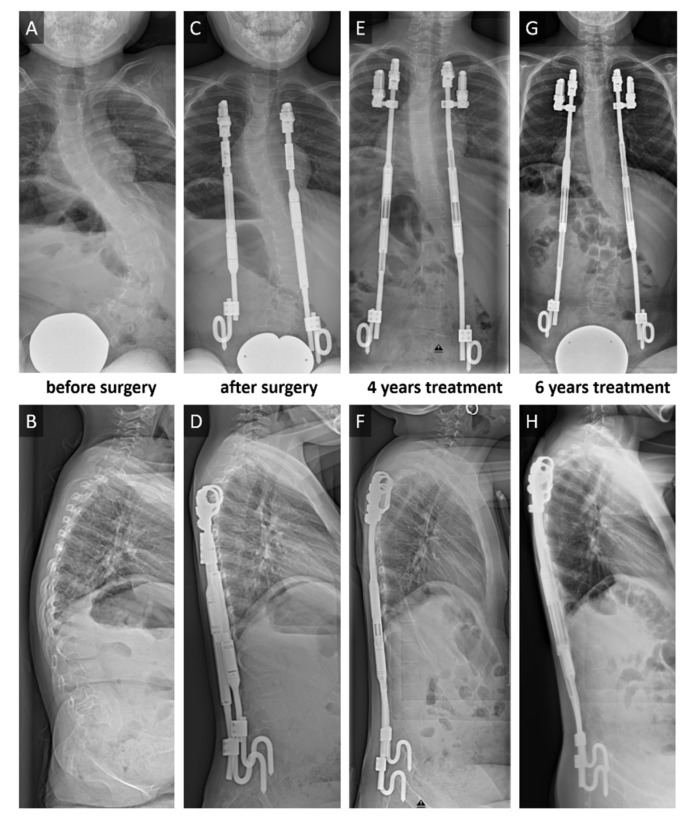
Bilateral rib-to-pelvis fixation with magnetically controlled growing rods (MCGR) in a 5-year-old spinal muscular atrophy type II girl with progressive scoliosis. Posterior anterior (p.a.) (**A**) and lateral (**B**) sitting radiographs before the surgical intervention. Deformity correction (**C/D**) after surgical treatment. Implant exchange after maximal distraction after 3 years of treatment. 4-year follow-up (**E/F**) with second MCGRs. 6-year follow-up (**G/H**) with fully distracted rods.

While the overall impression of this method, which has been used for several decades now, is positive, there is some doubt that the benefits outweigh the complications. GFSI is frequently associated with heterotopic ossification and autofusion of the spine [3,4,5]. One of the concerns raised in the discussion is the effect of GFSI on the morphology of vertebrae and discs [6]. The evaluation of such changes is somewhat difficult because of several factors, such as a heterogeneous EOS population with many underlying diseases, genetic predispositions, multiple additional problems and disabilities. In addition, measurements of morphologic changes, i.e., vertebral or disc shape, or even estimation of biomechanical properties like stiffness of the spine, have mostly been based on plain X-rays, with all the limitations that two-dimensional imaging have when attempting the evaluation of three-dimensional phenomena [7]. Hasler et al. [6] have described some of the changes seen in lumbar vertebrae on radiographs after GFSI treatment and Lippross et al. [8] reported reduced vertebral body volume using magnetically controlled growing rods (MCGR). To our knowledge, few data have been published on intervertebral disc changes after GFSI treatment [9] and no data after MCGRs, despite disc mobility ultimately determining spinal flexibility. To shed light on some aspects of the emerging technique of MCGRs, we conducted a prospective study on a homogenous cohort of patients with spinal muscular atrophy (SMA) and spinal deformity. Almost all SMA patients suffer from EOS, and MCGRs have become a standard treatment for many [10,11]. This investigation evaluates intervertebral disc volume, disc height and disc degeneration of children before and after an average of 5.1 years of MCGR treatment in comparison with age- and disease-matched controls without surgery. Therefore, this study design allows us to examine solely the effect of MCGRs (Figure 1) on intervertebral discs in children with spinal muscular atrophy (SMA).

## 2. Materials and Methods

After ethics committee approval, a prospective non-randomized cohort study of 33 scoliotic non-ambulatory children with SMA type II was performed.

Cohort I (*n* = 17) were SMA children, who had received bilateral MCGRs with rib (ribs 2 to 4) to pelvis fixation at an average age of 8.3 (+/−1.4) years. Repeated magnetically controlled lengthening procedures with five mm per side were performed every three months. If the MCGR implant failed (*n* = 4) or infection occurred (*n* = 1), the implant was replaced, and the patients were kept in our study. Cohort II (*n* = 16) consisted of juvenile SMA patients who initially presented with severe spinal deformity without any prior surgical spine intervention. Late presentation was either due to refugee status or because of fear of surgical treatment.

**Figure 2:**
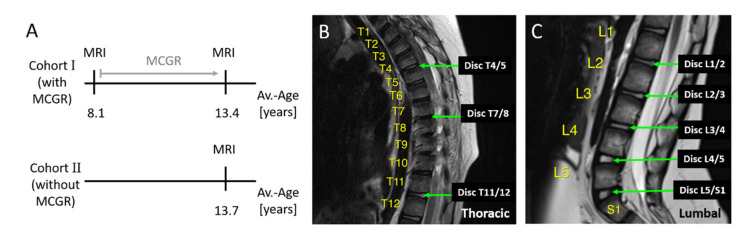
Cohorts and analyzed intervertebral discs. Cohort I had MRI investigations before and after on average 5.1 years of MCGR treatment. Cohort II had an MRI investigation at an average age of 13.7 years thus matching the late MRI exam of cohort I (**A**). On MRI scans intervertebral discs between thoracic vertebra 4 and 5 (T4/5), 7 and 8 (T7/8) and 11 and 12 (T11/12) were analyzed (**B**) as well as all intervertebral discs in the lumbar region (**C**).

Cohort I received two magnetic resonance imaging (MRI) examinations, one before the initial GFSI surgery at an average age of 8.1 (+/−1.4) years and the other after the removal of implants as a preparation for definite spinal fusion (average age 13.4 +/− 1.7 years). MCGRs were removed in all children, on average, 9.6 weeks (range 3–20) prior to definitive spinal fusion to minimize the risk of implant infection. Accordingly, cohort II received an MRI scan before spinal fusion at an average age of 13.7 (+/−2.6) years (Figure 2A). MRI examinations were performed using a 3-Tesla Trio Tim MRI Scanner (Siemens Healthcare GmbH, Erlangen, Germany) or Prisma Fit MRI Scanner (Siemens Healthcare GmbH, Erlangen, Germany) with 3 mm, 3.5 mm or 4 mm slice thickness.

On 49 MRI exams (33 cohort I and 16 cohort II), intervertebral discs between the thoracic vertebra (T) 4 and T5, T7 and T8, T11 and T12 (Figure 2B), and all lumbar intervertebral discs between lumbar vertebra (L) 1 and sacral vertebra (S) 1 (Figure 2C) were analyzed using the software OsiriX Lite^®^ (Pixmeo, Geneva, Switzerland). Thoracic intervertebral discs T4/5, T7/8 and T11/12 were chosen, because significant vertebral body volume changes and decreased vertebral depth were observed after MCGR treatment in T7 to L5, while T1 to T6 were not affected. Assuming that vertebral body volume and intervertebral disc volume are dependent on each other, we analyzed T4/5 (assuming no degenerative changes), T7/8 (to see if disc degeneration might precede vertebral body changes) and T11/T12 and below, because most changes can be expected in the lumbar area.

**Figure 3:**
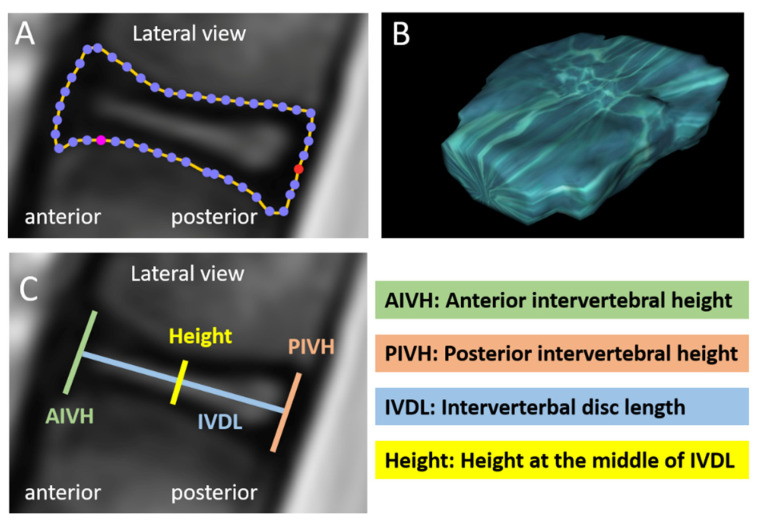
Measurements of intervertebral disc volume and intervertebral space dimensions. Intervertebral disc volume was determined by manual drawing of intervertebral disc circumference on each individual plane of the MRI scan (**A**) and subsequent software-based volume calculation (**B**). Intervertebral space dimensions were anterior intervertebral height (AIVH), posterior intervertebral height (PIVH), intervertebral disc length (IVDL) and height at the middle of IVDL. These parameters were measured on each individual plane of lateral MRI images (**C**) in order to use them for formula-based calculation of the intervertebral space volume (Figure 4).

**Figure 4:**
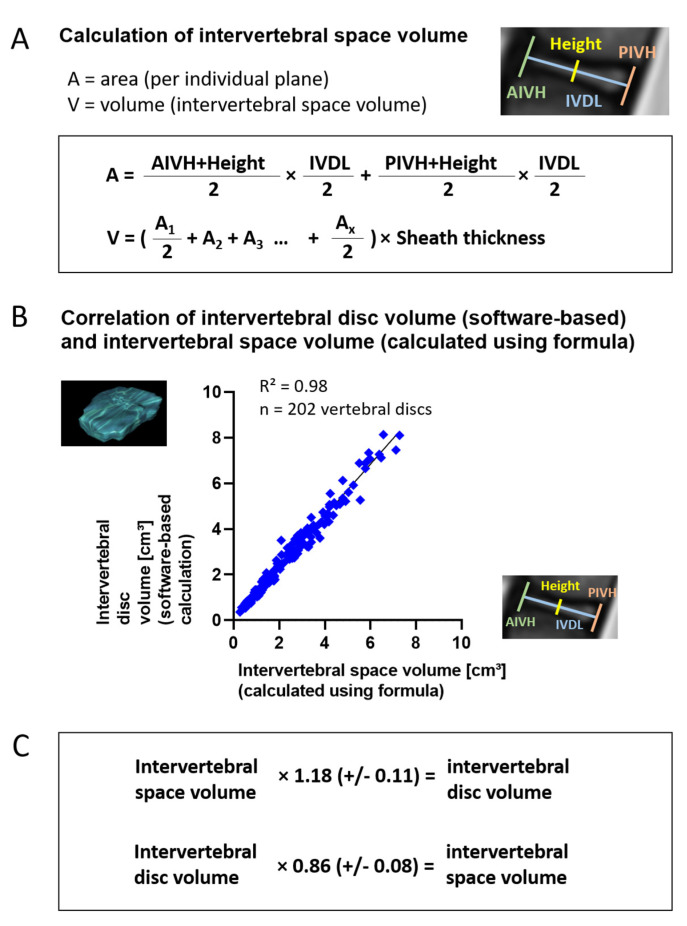
Calculation of intervertebral space volume and correlation with intervertebral disc volume. Intervertebral space dimensions measured as described in Figure 3 were used to calculate the intervertebral space volume using the formula depicted in (**A**). First, the area A of the intervertebral space was calculated for each individual plane using the measured lengths as depicted in Figure 3 and the Trapezoidal rule. Again, using the Trapezoidal rule, the areas of all planes (A1, A2, A3, … Ax with x being the number of planes) were subsequently multiplied by the sheath thickness of MRI scans to gain the total intervertebral space volume V. Correlation of intervertebral disc volume (determined by drawing of circumferences and software-based calculation) and intervertebral space volume (determined by landmark measurements and calculation using formula) for 202 intervertebral discs (T4/5, T7/8, T11/12, L1/2-L5/S1) revealed a strong correlation of both methods (R^2^ = 0.98) (**B**). On average, the intervertebral disc volume was larger by a factor of 1.18 than the intervertebral space volume, so that 1.18 (+/−0.11) may be applied as a correction factor to calculate the intervertebral disc volume from the intervertebral space volume, and a correction factor of 0.86 (+/−0.08) may be applied vice versa. (**C**). Given is mean +/− standard deviation (SD).

Intervertebral disc volume and intervertebral space volume were determined by two different approaches. The intervertebral disc volume was measured by the manual drawing of intervertebral disc circumference (Figure 3A) on all individual sagittal plains of the MRI scan (between 4 and 12 plains per patient) and subsequent software-based volume calculation (Figure 3B). The intervertebral space volume was determined by measuring distances between vertebral landmarks (Figure 3C) similar to Tunset et al. [12], calculating the area per plane and, subsequently, the volume from all area-measurements (Figure 4A). In order to compare both methods, volume measurements were done on 202 intervertebral discs using both approaches (Figure 4B).

Intervertebral disc degeneration (IDD) was graded by five independent investigators according to Pfirrmann et al. [13]. To exclude a possible bias, grading was performed in a blinded manner. Pfirrmann et al. [13] described a classification system of IDD graded I to V, with grade I being normal for lumbar intervertebral discs. In this study, all lumbar and some thoracic intervertebral discs (T4/5, T7/8, T11/12) were graded accordingly. Mean values of all investigators were used for calculation.

The acquired data were statistically analyzed using Excel Version 2016 (Microsoft Cooperation, Redmond, Washington, DC, USA) and Graph Pad Prism Version 6 (GraphPad Software Inc. San Diego, CA, USA). A paired *t*-test was used to compare paired data between the early and late time points within cohort I. An unpaired *t*-test was used to compare unpaired data between the cohorts at the late timepoint. For comparison of the Pfirrmann score, a Wilcoxon test was used to compare early and late patients with MCGRs and a Mann–Whitney test was used to compare patients after MCGR treatment at the late time point with age- and disease-matched untreated patients. The type of tests applied are also mentioned in the corresponding figure legends. For data analysis, only those intervertebral discs were considered that could be clearly measured and scored accordingly, thus explaining the different *n* numbers indicated in the figure legends. Statistical significance was defined with levels as *p* < 0.05 (*), *p* < 0.01 (**) and *p* < 0.001 (***).

## 3. Results

To assess the effect of MCGRs on intervertebral discs in scoliotic children, 49 MRI scans of 33 children were analyzed. In cohort I intervertebral disc changes could be analyzed after an average of 5.1 (+/−2.0) years of MCGR treatment. The second MRI of cohort I was age- and disease-matched to MRI exams of cohort II (Table 1). All of the 17 patients from Cohort I were used for the comparison to Cohort II, whereas, for the paired before and after analysis, only 16 patients from Cohort I were evaluated.

Spinal deformity of the frontal plane was more severe in untreated children (cohort II) in comparison to pre-treated individuals (cohort I) at the same age. Height, weight and BMI were comparable in both groups.

### 3.1. Intervertebral Disc Volume, Height and Depth

**Figure 5:**
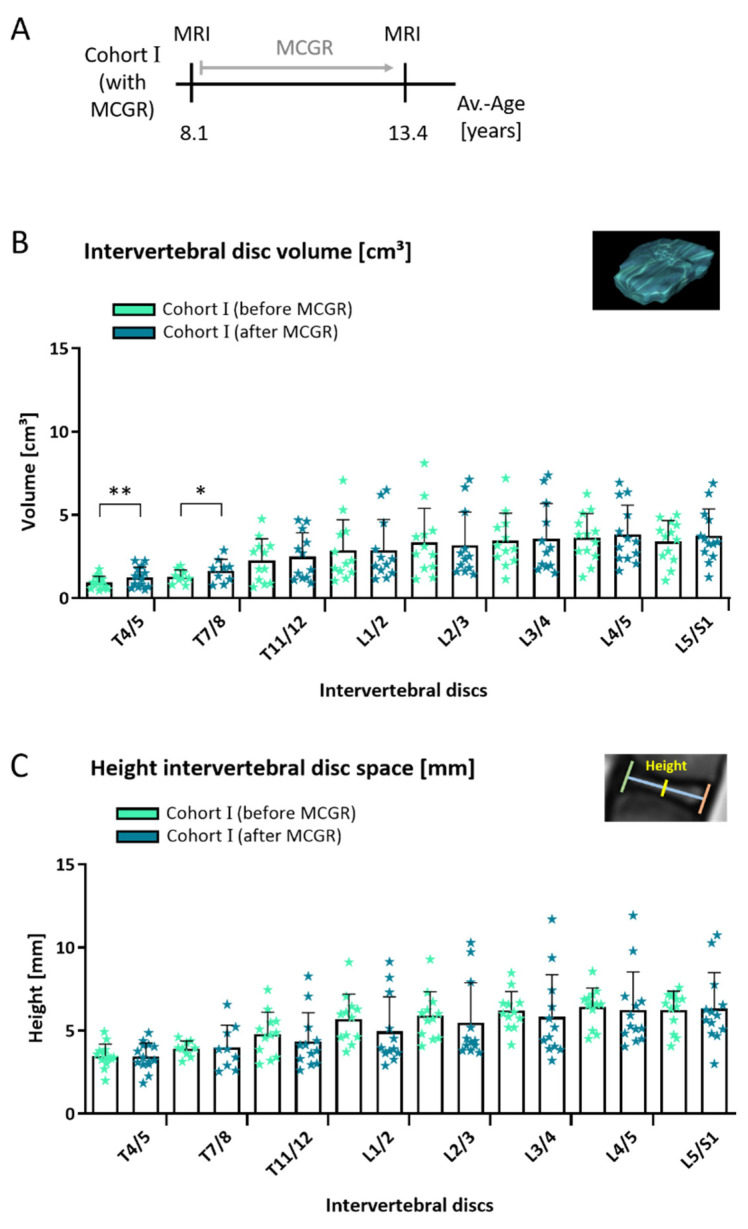
Intervertebral disc volume in children before and after 5.1 years of MCGR treatment. In cohort I, MRI measurements of children with spinal deformity were compared before and after 5.1 years of MCGR treatment (**A**). No significant changes in intervertebral disc volumes (**B**) and height of the intervertebral disc space (**C**) were seen after 5.1 years in the lower thoracic and lumbar spine. (B and C) Paired *t*-test; mean +/− SD; early (Cohort I before MCGR) and late (Cohort I after MCGR) time point: *n* = 14 (T4/5), *n* = 9 (T7/8), *n* = 12 (T11/12), *n* = 12 (L1/2), *n* = 12 (L2/3), *n* = 12 (L3/4), *n* = 13 (L4/5) and *n* = 13 (L5/S1); statistical level of significance *p* < 0.05 (*), *p* < 0.01 (**).

In cohort I (*n* = 16), there were no significant changes of intervertebral disc volume in the lower thoracic and lumbar spine after 5.1 years of MCGR treatment (Figure 5). The comparison of intervertebral space volumes led to equivalent results. Over time, an increased disc volume was seen in the upper and mid-thoracic area. Intervertebral disc height (Figure 5) and depth did not change within the follow-up period.

**Figure 6:**
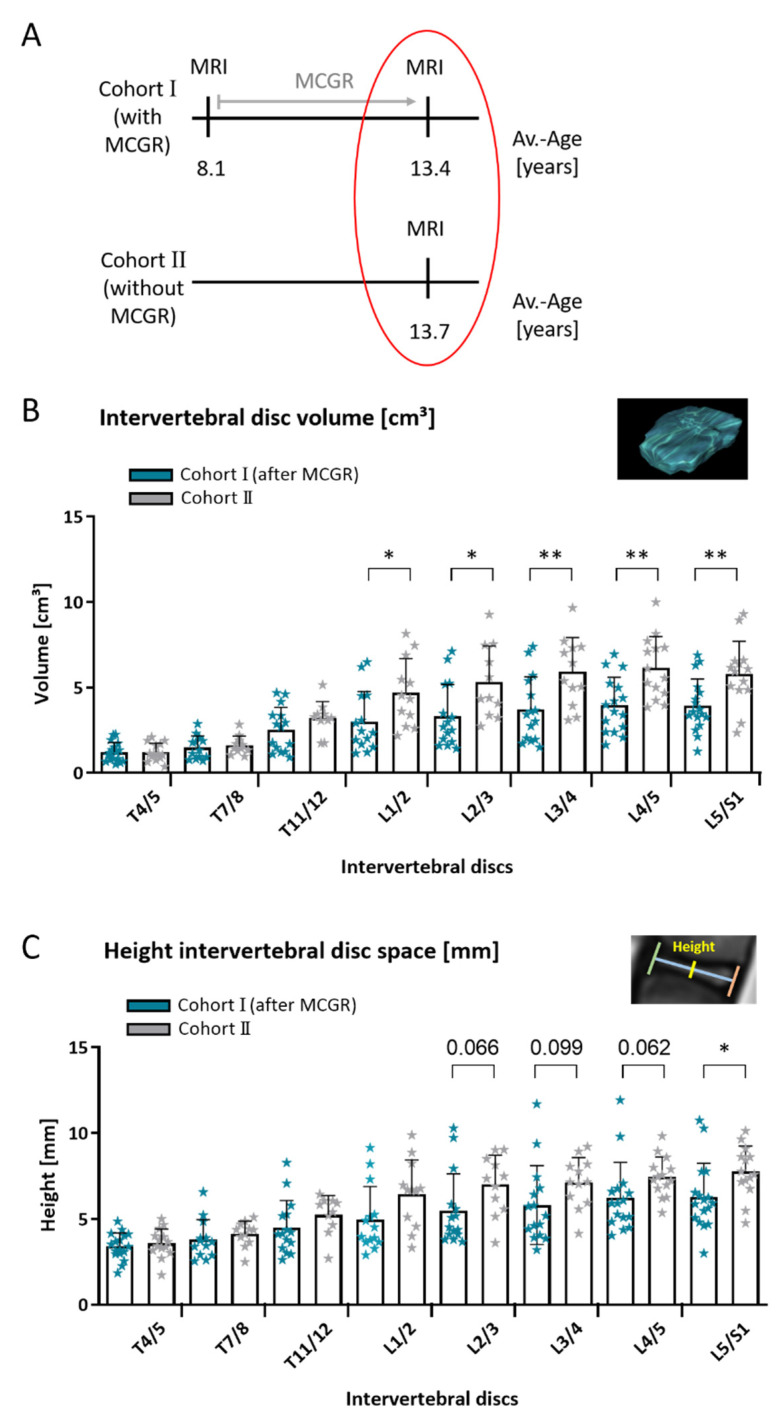
Intervertebral disc volume in age- and disease-matched children with and without MCGR. Comparing age- and disease-matched children with and without prior MCGR (**A**). Untreated children had significantly higher lumbar disc volumes in comparison to MCGR-treated children (**B**). Note the trend towards increased height of intervertebral disc space in untreated children (**C**). (**B**) and (**C**) Unpaired *t*-test; mean +/− SD; *n* = 17/14 (T4/5), 13/11 (T7/8), 15/10 (T11/12), 14/12 (L1/2), 15/11 (L2/3), 15/12 (L3/4), 16/14 (L4/5) and 17/14 (L5/S1) with prior MCGR (Cohort I after MCGR) and without prior MCGR (Cohort II) respectively; statistical level of significance *p* < 0.05 (*), *p* < 0.01 (**); for *t*-test results close to significance, *p*-values are given above the corresponding bars.

Comparing age- and disease-matched data of MCGR treated (Cohort I, *n* = 17) versus untreated patients, there was a significantly higher lumbar intervertebral disc volume in untreated children in comparison to treated children (Figure 6). Again, the comparison of intervertebral space volumes led to equivalent results. There was also a trend towards increased intervertebral disc height (Figure 6) and significantly increased intervertebral disc depth from T11/12–L5/S1 in untreated individuals.

### 3.2. Intervertebral Disc Degeneration

The Pfirrmann [13] score was used to determine degenerative disc changes evaluating 346 intervertebral discs by five investigators, in an independent and blinded manner. Inter-observer analysis revealed similar results and mean values were taken.

**Figure 7:**
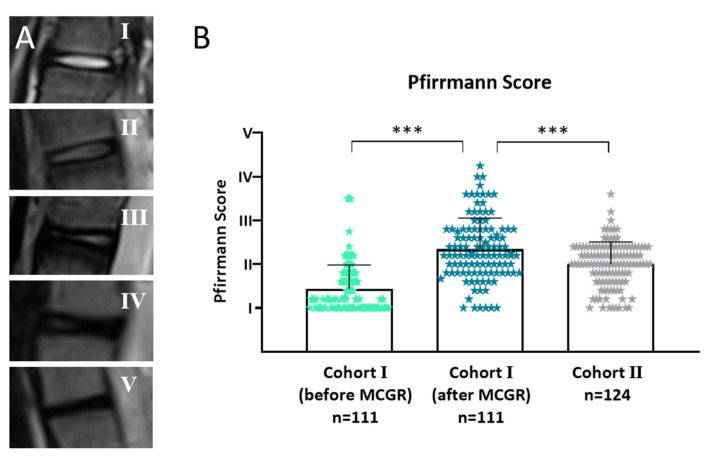
Intervertebral disc degeneration (IDD) measured with the Pfirrmann [13] score in 346 intervertebral discs. (**A**) Example images from the study population representing Pfirrmann scores I-V. (**B**) After an average of 5.1 years of MCGR treatment there was significantly more IDD in the same population. Age- and disease-matched patients without prior surgical treatment had significantly less IDD in comparison to pretreated individuals. Intervertebral discs were scored by five independent observers in a blinded manner and mean values from all observers are displayed. Cohort I (before MCGR) vs. Cohort I (after MCGR) Wilcoxon matched pairs test; Cohort I (after MCGR) vs. Cohort II Mann–Whitney test, *n*-numbers are given in the graph; included are *n* = 16/16/15 (T4/5), 14/14/15 (T7/8), 16/16/14 (T11/12), 13/13/16 (L1/2), 13/13/16 (L2/3), 13/13/16 (L3/4), 13/13/16 (L4/5), 13/13/16 (L5/S1), intervertebral discs from Cohort I (before MCGR), Cohort I (after MCGR) and Cohort II respectively; statistical level of significance *p* < 0.001 (***).

There was significantly more intervertebral disc degeneration (IDD) in cohort I after 5.1 years of MCGR treatment in comparison to MRI investigations before surgery (Figure 7). Age- and disease-matched patients (cohort I after MCGR versus cohort II) showed significantly more IDD after MCGR therapy in comparison to untreated individuals, despite more severe scoliosis in the latter group.

### 3.3. Volume Calculation

In order to test two methods of volume calculation, 202 intervertebral discs were measured by two approaches: (1.) by manual drawing of intervertebral disc circumferences on individual planes and subsequent software-based volume calculation (intervertebral disc volume) and (2.) by measuring distances between vertebral landmarks and calculating the volume in between vertebrae (intervertebral space volume) using the formula depicted in (Figure 4A). A strong correlation between the values gained with both methods was found with R^2^ = 0.98 (Figure 4B). On average, the intervertebral space volume (calculated using formula) was smaller than the intervertebral disc volume (software-based calculation) by 14.4% (+/−7.6). Therefore, the factor 1.18 (+/−SD 0.11) may be applied as a correction factor to calculate the intervertebral disc volume from the intervertebral space volume, and a correction factor of 0.86 (+/−SD 0.08) may be applied vice versa (Figure 4C). In this paper, data are displayed using the intervertebral disc volumes determined by manual drawing and subsequent software-based volume calculation. The same results were obtained using intervertebral space volumes based on formula calculation.

## 4. Discussion

Growth-friendly spinal implants (GFSI) have become a frequently applied technique to overcome the dilemma of severe and progressive deformity of the growing spine in early-onset scoliosis (EOS). It is accepted that the severity of the deformity determines pulmonary compromise and quality of life [14,15]. There are many promising reports, not least by our own group, that suggest a positive effect of GFSI on curve control and a better outcome at later definitive spinal fusion [1,11,16,17]. Yet, some argue that restricting the motion of the spine and thorax by GFSI may lead to increased rigidity or even autofusion, making a definitive surgical fusion more challenging, less effective or even unnecessary [3,7,18]. GFSI induced changes in the bony spine, i.e., the vertebrae, have been hypothesized for long but there are very few reports on actual measurements of spine morphology under GFSI treatment. Hasler et al. [6] have proposed an increase in vertebral height during treatment, which was measured on plain radiographs and is therefore susceptible to errors. Our own group has published data on reduced vertebral body volume in depth, below the level of T7, comparing untreated versus pre-treated children with MCGRs [8].

To our knowledge there are few data published on intervertebral disc changes during GFSI or MCGR treatment [9]. In this investigation, intervertebral discs of a prospectively followed cohort of children with spinal muscular atrophy (SMA) and progressive spinal deformity was evaluated before and after MCGR treatment and matched to untreated SMA children. The cohort is, therefore, homogenous to an extent that is hardly ever achieved in other studies involving EOS patients [9,19,20]. Furthermore, most children suffering from SMA are treated with GFSI if their life expectancy allows for this [21,22]. In fact, the prognosis of SMA has recently changed dramatically with the advent of gene therapy [23]. The comparably large number of SMA patients at our institution enables us to investigate and compare effects on spinal morphology by analysis of MRI scans that are routinely obtained before and after MCGR treatment and before definitive spinal fusion.

MRI is the primary diagnostic tool for analysis of soft tissue morphology. Here, we use MRI scans to investigate intervertebral disc degeneration (IDD) according to the well-established Pfirrmann method [13,24], graded by five independent observers (three of those being experienced clinicians) blinded with respect to the study population. While IDD is believed to be a primary factor related to back pain and increasing age, IDD in EOS must rather be seen as an indicator of reduced range of motion and spinal stiffness and/or scoliotic deformity [25,26]. Demirkiran et al. [26] found some disc degeneration after non-fusion spinal distraction in piglets, but postulated that growing rods did not impair disc health. Rong et al. [9] analyzed intervertebral disc space in heterogenous EOS children treated with dual growing rods. A decreased disc space was shown radiologically by the authors over time [9]. Several authors have also postulated spinal stiffness and/or autofusion through GFSI [4,7] and our findings support these observations as there is significantly more IDD after MCGR treatment in the same patients, as well as in comparison to age- and disease-matched controls. Additionally, disc height does not increase sufficiently over time (Figure 5) and has a trend towards lower heights in pre-treated individuals (Figure 6). Consequently, during MCGR treatment, intervertebral disc volume did not increase by age, as one would expect, and was also reduced in comparison to untreated age- and disease-matched individuals. Even though MCGRs were not directly attached to the spine [19,20], implants probably caused a reduction in motion, thus leading to reduced disc volume and degenerative changes. Scoliosis was more severe in untreated children than in MCGR-treated children. Scoliosis itself was proven as a risk factor of IDD [27,28,29]. However, in this population, prior MCGR therapy had a more severe effect on disc health than the actual degree of scoliosis. Rajasekaran et al. [29] postulated nutritional factors with alterations to diffusion and damage to the end plate as the primary mechanism of disc degeneration induced by mechanical stress, and Gervais et al. [30] focused on MRI signal intensity 3D distribution within the intervertebral disc as a tool for evaluation of disc degeneration. In this study, IDD was solely graded by the Pfirrmann score and no further perfusion data were obtained.

In addition to IDD grading, we used a software-based calculation technique to measure disc volumes. The method is established and can be applied to almost any MRI scan [31,32]. Our investigation showed a reduced disc volume, height and depth, pronounced in the lower spine, when MCGRs were applied. At first glance, this finding sounds controversial, as distraction would be thought to increase the overall height of spinal elements, including the intervertebral discs. However, motion, flexibility and weight bearing seem to be essential for healthy disc morphology and all these factors are influenced by GFSI treatment [9,29,33,34].

All MRI volume calculation techniques bear one critical problem, that is, slice thickness [31]. One of the reasons why MRI is still not used as a gold standard in bone imaging is that the thickness of the slices leads to some reduction in the resolution of the image. Disc volumes as investigated here are displayed as a continuous body, but this image is created from only 4 to 12 slices. Especially towards the edges of a disc, this method will always lead to some uncertainty about the real object. This uncertainty may be seen as a systematic error, as all of our measurements are affected by it. To reduce a possible bias, we have, therefore, used different methods to calculate (1.) intervertebral disc volume by using a standard open-source software to encircle disc circumferences and for software-based volume calculation and (2.) intervertebral space volume by measuring dimensions, i.e., height and diameter and using a mathematical formula. Even though all measurements suggest the same results, this point remains worth discussing.

## 5. Conclusions

In summary, this study analyzed, for the first time, the effect of MCGRs on intervertebral discs in EOS patients. Reduced intervertebral disc volume and more pronounced degenerative changes were seen after 5.1 years of MCGR treatment in comparison to age- and disease-matched controls. Despite the good corrective results of MCGRs, this aspect must be considered with respect to the reduced spinal flexibility, autofusion and planning of fusion levels.

## Figures and Tables

**Table 1:** Patient demographics.

	Age/Gender (f = Female/ m = Male)	Height/Weight/BMI	Spinal Deformity (Cobb Angle in °)
**Cohort I ** 1. MRI (*n* = 16) (before GFSI)	8.1 (+/−1.4) years(6f/10 m)		70.1 (+/−22.6) before initial surgery 34.0 (+/−15.4) after initial surgery
2. MRI (*n* = 17) (after GFSI)	13.4 (+/−1.7) years (7f/10 m)	height 146 (+/−8) cm weight 39.1 (+/−9.5) kg BMI 18.4 (+/−3.9)	65.8 (+/−20.2) after GFSI treatment prior to spinal fusion
MCGR index surgery age 8.3 (+/−1.4) years			
**Cohort II ** (*n* = 16) No surgical treatment	13.7 (+/−2.6) years (11f/5 m)	height 147 (+/−11) cm weight 42.6 (+/−14.0) kg BMI 19.6 (+/−6.2)	89.6 (+/−34.9)

MRI = magnetic resonance imaging; MCGR = magnetically controlled growing rods; BMI = body mass index.

## Data Availability

The data presented in this study are available on request from the corresponding author.

## References

[1] Bekmez S., Afandiyev A., Dede O., Karaismailoğlu E., Demirkiran H.G., Yazici M. (2019). Is Magnetically Controlled Growing Rod the Game Changer in Early-onset Scoliosis? A Preliminary Report. J. Pediatr. Orthop..

[2] Carbone M., Vittoria F., Del Sal A. (2019). Treatment of early-onset scoliosis with growing rods in patients with neurofibromatosis-1. J. Pediatr. Orthop. B.

[3] Cahill P.J., Marvil S., Cuddihy L., Schutt C., Idema J., Clements D.H., Antonacci M.D., Asghar J., Samdani A.F., Betz R.R. (2010). Autofusion in the Immature Spine Treated With Growing Rods. Spine.

[4] Groenefeld B., Hell A.K. (2013). Ossifications after Vertical Expandable Prosthetic Titanium Rib Treatment in Children with Thoracic Insufficiency Syndrome and Scoliosis. Spine.

[5] Bouthors C., Izatt M.T., Adam C.J., Pearcy M.J., Labrom R.D., Askin G.N. (2018). Minimizing Spine Autofusion With the Use of Semiconstrained Growing Rods for Early Onset Scoliosis in Children. J. Pediatr. Orthop..

[6] Hasler C.C., Studer D., Büchler P. (2015). Metamorphosis of human lumbar vertebrae induced by VEPTR growth modulation and stress shielding. J. Child. Orthop..

[7] Sankar W.N., Skaggs D.L., Yazici M., Johnston C.E., Shah S.A., Javidan P., Kadakia R.V., Day T.F., Akbarnia B.A. (2011). Lengthening of Dual Growing Rods and the Law of Diminishing Returns. Spine.

[8] Lippross S., Grages A., Lueders K.A., Braunschweig L., Austein F., Tsaknakis K., Lorenz H.M., Hell A.K. (2021). Vertebral body changes after continuous spinal distraction in scoliotic children. Eur. Spine J..

[9] Rong T., Shen J., Kwan K., Zhang J., Wang Y., Li S., Li Z., Chen C., Lin Y., Tan H. (2019). Vertebral Growth Around Distal Instrumented Vertebra in Patients With Early-Onset Scoliosis Who Underwent Traditional Dual Growing Rod Treatment. Spine.

[10] Wijngaarde C.A., Brink R.C., De Kort F.A., Stam M., Otto L.A.M., Asselman F.-L., Bartels B., Van Eijk R.P., Sombroek J., Cuppen I. (2019). Natural course of scoliosis and lifetime risk of scoliosis surgery in spinal muscular atrophy. Neurology.

[11] Hell A.K., Braunschweig L., Tsaknakis K., Von Deimling U., Lüders K.A., Hecker M., Lorenz H.M. (2020). Children With Spinal Muscular Atrophy With Prior Growth-Friendly Spinal Implants Have Better Results After Definite Spinal Fusion in Comparison to Untreated Patients. Neurosurgery.

[12] Tunset A., Kjaer P., Chreiteh S.S., Jensen T.S. (2013). A method for quantitative measurement of lumbar intervertebral disc structures: An intra- and inter-rater agreement and reliability study. Chiropr. Man. Ther..

[13] Pfirrmann C.W.A., Metzdorf A., Zanetti M., Hodler J., Boos N. (2001). Magnetic Resonance Classification of Lumbar Intervertebral Disc Degeneration. Spine.

[14] Campbell M., Matsumoto H., Hilaire T.S., Roye B.D., Roye D.P., Vitale M.G. (2020). Burden of care in families of patients with early onset scoliosis. J. Pediatr. Orthop. B.

[15] Roye B.D., Simhon M.E., Matsumoto H., Garg S., Redding G., Samdani A., Smith J.T., Sponseller P., Vitale M.G., Children’s Spine Study Group (2020). Bigger is better: Larger thoracic height is associated with increased health related quality of life at skeletal maturity. Spine Deform..

[16] Ridderbusch K., Rupprecht M., Kunkel P., Hagemann C., Stücker R. (2017). Preliminary Results of Magnetically Controlled Growing Rods for Early Onset Scoliosis. J. Pediatr. Orthop..

[17] Campbell R.M., Hell-Vocke A.K. (2003). GROWTH OF THE THORACIC SPINE IN CONGENITAL SCOLIOSIS AFTER EXPANSION THORACOPLASTY. J. Bone Jt. Surg. Am. Vol..

[18] Lattig F., Taurman R., Hell A.K. (2016). Treatment of Early-Onset Spinal Deformity (EOSD) With VEPTR. Clin. Spine Surg..

[19] Lebon J., Batailler C., Wargny M., Choufani E., Violas P., Fron D., Kieffer J., Accadbled F., Cunin V., De Gauzy J.S. (2017). Magnetically controlled growing rod in early onset scoliosis: A 30-case multicenter study. Eur. Spine J..

[20] Subramanian T., Ahmad A., Mardare D.M., Kieser D.C., Mayers D., Nnadi C. (2018). A six-year observational study of 31 children with early-onset scoliosis treated using magnetically controlled growing rods with a minimum follow-up of two years. Bone Jt. J..

[21] Lorenz H.M., Braunschweig L., Eberhardt I.M., Tsaknakis K., Hell A. (2019). Operative „No-touch“-Techniken zur Korrektur kindlicher Skoliosen. Oper. Orthopädie Traumatol..

[22] Hell A.K., Groenefeld K., Tsaknakis K., Braunschweig L., Lorenz H.M. (2018). Combining Bilateral Magnetically Controlled Implants Inserted Parallel to the Spine With Rib to Pelvis Fixation. Clin. Spine Surg..

[23] Szabó L., Gergely A., Jakus R., Fogarasi A., Grosz Z., Molnár M.J., Andor I., Schulcz O., Goschler Á., Medveczky E. (2020). Efficacy of nusinersen in type 1, 2 and 3 spinal muscular atrophy: Real world data from Hungarian patients. Eur. J. Paediatr. Neurol..

[24] Urrutia J., Besa P., Campos M., Cikutovic P., Cabezon M., Molina M., Cruz J.P. (2016). The Pfirrmann classification of lumbar intervertebral disc degeneration: An independent inter- and intra-observer agreement assessment. Eur. Spine J..

[25] Sitte I., Kathrein A., Pfaller K., Pedross F., Klosterhuber M., Lindtner R.A., Zenner J., Ferraris L., Meier O., Koller H. (2013). Morphological Differences in Adolescent Idiopathic Scoliosis. Spine.

[26] Demirkiran G., Yilgor C., Ayvaz M., Kosemehmetoglu K., Daglioglu K., Yazici M. (2014). Effects of the Fusionless Instrumentation on the Disks and Facet Joints of the Unfused Segments. J. Pediatr. Orthop..

[27] Huber M., Gilbert G., Roy J., Parent S., Labelle H., Périé D. (2016). Sensitivity of MRI parameters within intervertebral discs to the severity of adolescent idiopathic scoliosis. J. Magn. Reson. Imaging.

[28] Veres S.P., Robertson P.A., Broom N.D. (2010). ISSLS Prize Winner: How Loading Rate Influences Disc Failure Mechanics. Spine.

[29] Rajasekaran S., Vidyadhara S., Subbiah M., Kamath V., Karunanithi R., Shetty A.P., Venkateswaran K., Babu M., Meenakshi J. (2010). ISSLS Prize Winner: A Study of Effects of In Vivo Mechanical Forces on Human Lumbar Discs With Scoliotic Disc as a Biological Model. Spine.

[30] Gervais J., Périé D., Parent S., Labelle H., Aubin C.-E. (2012). MRI signal distribution within the intervertebral disc as a biomarker of adolescent idiopathic scoliosis and spondylolisthesis. BMC Musculoskelet. Disord..

[31] Ross J.S., Modic M.T. (1992). Current assessment of spinal degenerative disease with magnetic resonance imaging. Clin. Orthop. Relat. Res..

[32] Louzada L.L., Morato T.N., Almeida R.E.F., Camargos E.F., Nóbrega O.T., Farage L. (2016). OsiriX™ as a feasible tool for in office manual hippocampal volumetry in the elderly: A technical note. Geriatr. Gerontol. Aging.

[33] Gullbrand S.E., Peterson J., Ahlborn J., Mastropolo R., Fricker A., Roberts T.T., Abousayed M., Lawrence J.P., Glennon J.C., Ledet E.H. (2015). ISSLS Prize Winner. Spine.

[34] Gullbrand S.E., Kim D.H., Ashinsky B.G., Bonnevie E.D., Smith H.E., Mauck R.L. (2020). Restoration of physiologic loading modulates engineered intervertebral disc structure and function in an in vivo model. JOR Spine.

